# Health care workers’ knowledge on identification, management and treatment of snakebite cases in rural Malawi: A descriptive study

**DOI:** 10.1371/journal.pntd.0010841

**Published:** 2022-11-21

**Authors:** Moses Banda Aron, Chiyembekezo Kachimanga, Benno Kreuels, Bright Mailosi, Clara Sambani, Beatrice Lydia Matanje, Joerg Blessmann, Mwayi Chunga, Grace Momba, Enoch Ndarama, Dzinkambani Moffat Kambalame, Emilia Connolly, Anat Rosenthal, Fabien Munyaneza

**Affiliations:** 1 Partners In Health / Abwenzi Pa Za Umoyo, Neno, Malawi; 2 Research Group Snakebite Envenoming, Bernhard Nocht Institute for Tropical Medicine, Hamburg, Germany; 3 Section for Tropical Medicine, I. Department of Medicine, University Medical Center Hamburg, Germany; 4 Department of Research, Ministry of Health, Lilongwe, Malawi; 5 Neno District Health Office, Ministry of Health, Neno, Malawi; 6 Division of Pediatrics, University of Cincinnati College of Medicine, Cincinnati, Ohio, United States of America; 7 Division of Hospital Medicine, Cincinnati Children’s Hospital Medical Center, Cincinnati, Ohio, United States of America; 8 Department of Health Policy and Management, Ben-Gurion University of the Negev, Beersheba, Israel; Fundação de Medicina Tropical Doutor Heitor Vieira Dourado, BRAZIL

## Abstract

Snakebite envenoming remains a public health threat in many African countries, including Malawi. However, there is a shortage of literature on the knowledge of Health Care Workers (HCWs) and the prevalence of snakebite cases in Malawi. We interviewed HCWs in Neno District to assess their knowledge of snake identification and management of snakebites. We further reviewed patient registers from 2018 to 2021 in all 15 health facilities in the district. We used descriptive statistics to characterize the survey population, knowledge, snake antivenom (SAV) administration, and snake identification. Using "shapefiles" from Open Street Maps, we mapped villages with snakebite cases. Of the 105 HCWs interviewed, 58% were males, and 60% had worked for less than five years. The majority (n = 93, 89%) reported that snakebite envenoming was a problem in the district. Among the clinicians, 42% said they had prescribed SAV previously, while among nurses, only 26% had ever administered SAV. There were discrepancies among clinicians regarding the dosing of snake antivenom. Significant gaps in knowledge also existed regarding snake identification. While two-thirds of HCWs could correctly name and identify venomous snake species, most (> 90%) failed for non-venomous snakes. Most (n = 100, 95%) reported that snakebite victims visit traditional healers more than the hospital. Between 2018 and 2021, the Neno District registered 185 snakebites with a yearly average of 36 cases per 100,000 population. Fifty-two percent (n = 97) were treated as an inpatient; of these cases, 72% were discharged in less than three days, and two died. More snakebite cases were recorded in the eastern part of the district. Significant knowledge gaps exist among HCWs in Neno regarding prescription and administration of SAV and snake identification, which likely challenges the quality of services offered to snakebite victims.

## Author summary

Knowledge about management of snakebite envenoming and the availability of effective antivenom against the relevant venomous snake species are a “sine qua non” for a favorable outcome. There is no published literature addressing snakebite incidence and knowledge of health care workers in management of snakebite patients in Malawi. The present study investigated the number of snakebite cases managed at health facilities in Neno District in Southern Malawi between 2018 and 2021 and evaluated knowledge of health care workers about management of snakebite envenoming and the local snake fauna. Although the majority of interviewees considered snakebite a major problem, their knowledge in management of snakebite cases was not sufficient. Incidence of healthcare encounters due to snakebites in Neno District was calculated at 36 per 100,000 population. This is most likely a significant underestimation as treatment of victims by traditional healers in their village seems to be common. The study shows that further training of health care workers and more data on the epidemiology and clinical consequences of snakebites in Malawi are urgently needed.

## Introduction

Snakebites are one of the most overlooked Neglected Tropical Diseases (NTDs) [1] but pose a substantial public health threat in many tropical and subtropical countries worldwide. An estimated 5.5 million snakebites occur yearly, resulting in over 1.8 million cases of envenoming [2,3]. Snakebite envenoming causes considerable morbidity and mortality worldwide, with the highest burden in Asia, sub-Saharan Africa, and Central- and South America [1,3–5]. Everyone in these regions is at risk of being bitten by snakes. However, rural agricultural workers, herders, fishermen, hunters, and people living in poorly constructed houses bear a higher risk [6,7]. In 2017, snakebite envenoming was re-added to the list of NTDs following its removal in 2013 [1,4].

A snake-human conflict has existed for millennia. Some snakes inject into or spray venom at their victims, resulting in potentially life-threatening situations [8]. Approximately 81,000–138,000 snakebite victims die each year and many more suffer from long-term complications such as deformities, contractures, amputations, visual impairment, renal complications, and psychological distress [5,9,10]. High-quality snake antivenom (SAV) is the most effective treatment to prevent and reverse most effects of venomous snakebites. SAVs are species-specific and should only be administered intravenously [11–13]. SAVs are on the WHO list of essential medicines and should be part of any primary health care package where snakebites occur [7]. While SAVs are available in limited quantities in endemic settings in Asia and Latin America, for many years, there have been significant challenges with a reliable supply of effective products, costs, and adherence to the cold chain within Sub-Saharan Africa [12,14].

Across Sub-Saharan Africa, the number of people treated for snakebites in health facilities is estimated at 315,000 cases per year, with over 9,000 amputations and 7,000 deaths [15]. These numbers are likely to underestimate the actual situation due to the under-reporting of cases. In Malawi, a low-income country in southern Africa, there is a complete absence of literature on the estimated number of snakebites and treatment. A systematic review of 14 studies showed that most snakebite victims do not seek medical care at healthcare centers or hospitals due to distance to a health facility, financial constraints, and lack of availability of SAVs [16–18]. Studies done in rural Cameroon, Myanmar, Rwanda, and Ghana have also shown that traditional medicine is preferred and more trusted to cure snakebites than”western” bio-medicine [19–23].

Delayed treatment of snakebite patients is associated with increased mortality and morbidity. Health care workers’ (HCWs’) knowledge and compliance with standardized protocols for diagnosis and treatment remain crucial for managing snakebites [24–27]. Studies performed in Cameroon, Nigeria, and Lao PDR have shown that the level of knowledge among HCWs on snakebites remains poor [15,28,29]. In Bhutan, a study found that only 23% of HCWs had an adequate understanding of the management of snakebite envenoming, while in Ghana, a study showed a slightly higher level of knowledge at 48.2% [24,27]. While most HCWs are not herpetologists, identification of snake species and correct treatment of bites rely on healthcare workers to probe the history of the victims and their clinical signs and symptoms [30–32]. In many health facilities across Sub Saharan Africa, snakebite is not documented systematically [33].

Malawi is home to at least 66 different types of snakes, of which 11 are considered medically relevant and potentially deadly, while another five species are capable of inflicting extremely painful bites [34]. Malawi’s warm temperatures, mountainous forests, and lowlands with humid shrubs and forest covers provide an inviting habitat for snakes. The population’s dependence on subsistence farming, wood, and charcoal as a source of energy for cooking makes snake-human interactions common. While SAVs are on the WHO list of essential medicines, they are not part of Malawi’s essential medication list within the Essential Health Package. SAV availability is restricted to a few secondary and tertiary level health care facilities across the country and remains erratic [7,35]. There is a lack of data on the incidence of snakebites in Malawi and poor systematic documentation at facilities. Furthermore, there are no studies about the knowledge of HCWs on snakebite management. To understand knowledge and quantify snakebite cases, we assessed HCW’s knowledge of snake identification and snakebite treatment and undertook a retrospective review of snakebite cases documented in Neno District, Malawi.

## Methods

### Ethics statement

Ethical clearance was obtained from the Neno District Health Research Committee and the Institutional Review Board of Malawi National Health Science Research Committee (NHSRC/21/10/2816; dated: 26 November 2021). We obtained oral informed consent from the health care workers who participated in the study and were at liberty to withdraw from the study at any time. For review of the health facility registers which include data of snakebite cases of all age group, no consent was required as it was secondary data. Data were de-identified before analysis and kept securely by the investigator.

### Study design and setting

We conducted a cross-sectional study by administering a survey to HCWs between 21 December 2021 and 28 February 2022 and reviewed inpatient and outpatient registers for reported snakebites between 2018 and 2021 in Neno District, Malawi. In reporting, we followed Strengthening the Reporting of Observational Studies in Epidemiology (STROBE) guidelines (S1 STROBE Checklist) [36].

### The STROBE checklist

Neno District is located in the southwest part of the country and does not have tarmac roads in most parts of the district, making it very hard to be accessed during the rainy season [37]. The district can be divided into two sections in terms of topography: the western side which is primarily mountainous and the eastern side which is mostly flat lowland with shrub type vegetation.

The district has 15 health facilities that provide primary and secondary care to its residents. Neno District Hospital, five health centers (Dambe, Nsambe, Matandani, Neno Parish, and Margareta), and one dispensary (Ligowe) covering the western side. Lisungwi Community Hospital, three health centers (Luwani, Chifunga, Matope), and four dispensaries (Midzemba, Zalewa, Nkula, and Tedzani) covering the eastern side. Tedzani is co-shared facility between the Blantyre District Health Office and Neno District Health office regarding administration and management. All tertiary level health care services are provided through referral to Queen Elizabeth Central Hospital in Blantyre, which ensues a road journey of more than 3 hours [37].

In 2021, the Neno District had an estimated population of 147,272 [38]. While 61.7% of Malawi’s population is multidimensionally poor, the disparity is even higher in rural areas like Neno (over 70%) [39]. Neno residents are subsistence farmers and mainly depend on rain-fed agriculture. Annual rainfall of the Neno District ranges from 500mm to 1,200mm, with January being the wettest month and October the driest. Considering that only 4.5% of the population in Neno has electricity, almost everyone depends on wood and charcoal as a source of energy for cooking [37,40].

### Study population

**Fig 1:**
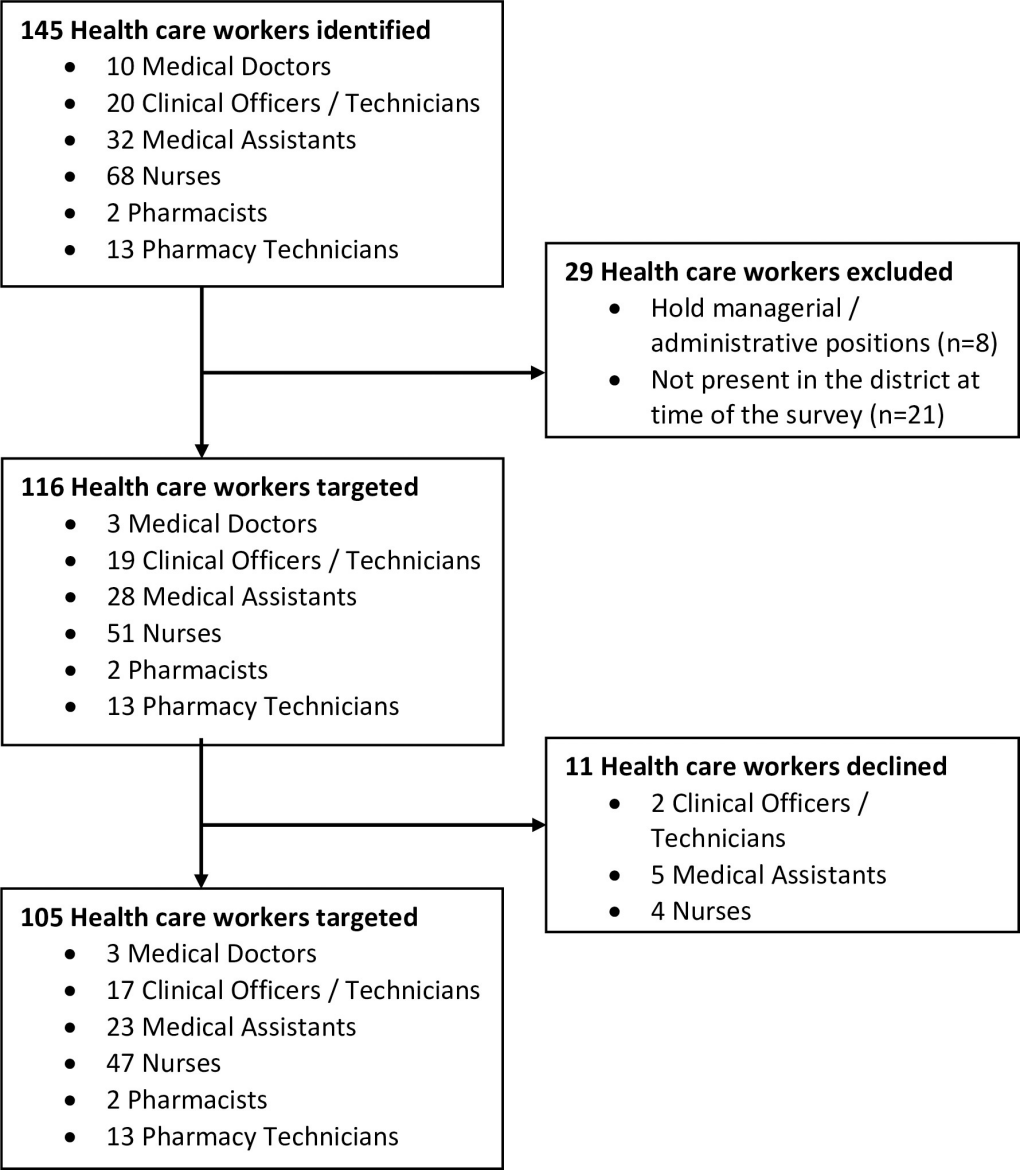
Health care workers targeted for snakebite survey in Neno District in 2021–2022.

At the time of the survey, we identified 145 HCWs providing services at the two secondary care hospitals and 13 primary health centers in Neno District. However, we excluded a total of 29 healthcare workers as they were leaving the district or not providing care to patients. Of the remaining 116 healthcare workers, 105 were willing to respond to the questionnaire (Fig 1).

In Malawi, medical doctors are trained for a minimum of 5 years plus an internship of 1.5 years and are awarded a Bachelor of Medicine and Bachelor of Surgery (MBBS) degree. Clinical officers/technicians are trained for three years of classwork and one year of internship and are awarded a diploma in clinical medicine. Medical assistants are trained for two years and one year of internship and are awarded a certificate in clinical medicine. Nurses complete a four-year Bachelor’s degree, a three-year diploma, or a two-year certification. Pharmacists complete a four-year Bachelor’s degree and pharmacy technicians are trained for a 3- and 2-years diploma and certificate, respectively.

For the retrospective snakebite case survey, we included all snakebite patients from facility registers who sought care from Neno District health facilities between January 2018 and December 2021.

### Data collection

We developed a questionnaire by reviewing the available literature on knowledge related to snakes and snakebite management (S1 Table) [24,27,41,42]. The survey questionnaire for HCWs had information on i) socio-demographic characteristics, including sex, age, current place of practice, and years of experience, ii) knowledge of snakebite antivenom and administration, iii) snake identification by pictures of snakes where participants were asked to identify the name of the snake in English or Chichewa (Malawi’s official languages) and whether it is venomous or non-venomous (S2 Table). The venomous snakes included puff adder [*Bitis arietans*], Black Mamba [*Dendroaspis polylepis*], Oates’ vine twig [*Thelotornis oastesi*], and Mozambique spitting cobra [*Naja mossambica*]. Non-venomous snakes included Common house snake [*Boaedon capensis*] and Spotted bush snake [*Philothamnus semivariegatus*] [34,43]. These are common snakes in Malawi and known to be involved in snakebite. We utilized open-ended questions to solicit prescription and administration of SAV protocols and practice and why snakebites are considered a problem in the Neno District. Additionally, we asked if HCWs think that snakebite victims visit local healers before they reach the hospital and why this may be the case.

The questionnaire was programmed into the CommCare application and installed on a tablet computer. CommCare is an easily customizable, open-source mobile platform that supports frontline workers in low-resource communities to collect data [44]. We trained two research assistants on the tools, one to administer the questionnaire to HCWs and another to review the health facility registers. For HCWs, we issued the questionnaire face to face with observance of COVID-19 precautionary measures. We pre-tested the questionnaire with six HCWs (3 medical assistant, 1 clinical officer and 2 nurses) who were leaving the district and ensured that the questions were clear and that understanding of the questions was uniform.

We reviewed all snakebite cases from outpatient and inpatient paper registers between January 2018 and December 2021 across all facilities in Neno District. Snakebite cases at Lisungwi community hospital and Neno District hospital were carefully reviewed to avoid double-counting patients referred from the health centers and dispensaries. For identified snakebite cases, we collected data on sex, age, traditional authority, name of the village, name of the health facility, date of presentation, type of care, outcome and for inpatient, number of days spent in the ward (S3 Table). We obtained “Base map and data from OpenStreetMap and OpenStreetMap Foundation” repository [45] and extracted Neno District boundaries.

### Data analysis

We extracted the HCW survey data from CommCare and entered snakebite case data into separate Microsoft Excel sheets. We imported data to R Software and utilized RStudio to clean and analyze the data. We used descriptive statistics such as counts and percentages for all categorical variables and median and Interquartile range (IQR) for all continuous variables. We defined three broad profession categories of HCWs considering the role played by each in snakebite management: (i) pharmacists and pharmacy technicians as "Pharmacy," (ii) nurses regardless of their level of education as "Nurses," and (iii) clinical officers/technicians, medical assistants and medical doctors as "Clinicians." Clinicians do the prescription and dosing; the pharmacy does the issuing, and nurses administer SAVs. We used semi-qualitative methods to present selected quotes reflecting patterns in the data from the open-ended questions. Quotes were chosen to better contextualize participants’ views on snakebites in Neno, how they prescribe and administer SAV and why they think that snakebite victims visit the traditional healers first before visiting the hospital. We categorized months of the year into the rainy season (November to April) and dry season (May to October). We used QGIS to create a map showing villages with snakebite cases treated at the health facilities across the district.

## Results

### Socio-demographic characteristics of health care workers

We interviewed 105 HCWs comprising of 47 nurses, 15 pharmacists and pharmacy technicians, and 43 "clinicians" (17 clinical officers/technicians, 23 medical assistants, and three medical doctors) with a response rate of 91%. In general, there were more males (58%) than females across the profession except among nurses, with 53% women, which is representative of Malawi’s health workforce. The median age was 30 years (IQR 22–38), and 61% of the HCWs were originally from rural areas. At the time of the survey, 58% of the HCWs were practicing at primary health centers or dispensaries. The majority of the HCWs (n = 63, 60%) had been practicing for not more than five years and 24 (22%) had been practicing for not more than one year; this was similar across all the professions (Table 1).

**Table 1:** Socio-demographic characteristics of study participants in Neno District.

		Profession		
Variable n (%)	Overall, N = 105	Nurses N = 47	Pharmacy N = 15	Clinicians N = 43
Sex				
Female	44 (42%)	25 (53%)	4 (27%)	15 (35%)
Male	61 (58%)	22 (47%)	11 (73%)	28 (65%)
Age (years)^1^	30.0 (27.0, 35.0)	29.0 (26.0, 33.0)	30.0 (27.5, 35.0)	31.0 (27.5, 36.0)
Originally from				
Rural	64 (61%)	29 (62%)	10 (67%)	25 (58%)
Urban	41 (39%)	18 (38%)	5 (33%)	18 (42%)
Place of practice				
Health center/dispensary	61 (58%)	26 (55%)	11 (73%)	24 (56%)
Hospital	44 (42%)	21 (45%)	4 (27%)	19 (44%)
Years of experience				
< = 1	24 (23%)	9 (19%)	3 (20%)	12 (28%)
2–5	39 (37%)	21 (45%)	5 (33%)	13 (30%)
6–10	22 (21%)	8 (17%)	5 (33%)	9 (21%)
Over 10	20 (19%)	9 (19%)	2 (13%)	9 (21%)

^1^Median (IQR)

### Snakebite training and experiences among health care workers in Neno, district

Most respondents indicated that snakebite envenoming is a problem in the district (n = 93, 89%), with the highest indication among clinicians (93%) (S4 Table). HCWs were concerned about the number of snakebite cases, as indicated in the survey’s open questions:

"*…Have had many patients presenting with snakebite including complications leading to amputations of limbs etc*…*"* Nurse at one of the health centers/dispensaries

"*…Our environment is bushy and with rocks which is a harbour of snakes; recently one of my house members was a victim of snakebite…*.*"* A pharmacy technician at one of the hospitals

Seventy three percent of HCWs (n = 77) indicated that they had received training on snakebite management. Of these, 66% and 16% received training as part of their formal training or during continuous professional development (CPD) respectively. Only 2 HCWs at Neno District hospital reported that snakebite management protocols are available. HCWs from all the facilities said they manage snakebites, with 62% admitting and treating, 33% giving first aid and referring the case to the secondary level hospital. Forty-six percent (n = 48) reported that they had treated or managed snakebite the previous year, but 47% (n = 49) of the respondents reported treating or managing fewer than 5 cases in the last year. HCWs said most patients were bitten on the legs (n = 97, 92%), with most bites occurring during the evening or night (n = 70, 67%).

Seventy-three percent (n = 77) of respondents reported that the most common cause of complications in snakebite victims was a delay in seeking treatment. However, 67% said there were no deaths due to snakebite in their facility in the past year. Interestingly, almost all HCWs (n = 100, 95%) said that snakebite victims prefer to go to a traditional healer more than to the hospital. They noted several reasons for this, ranging from beliefs and superstitions, unavailability of SAV at the facility, long distances, and poverty.

"*…Traditional healers are more accessible and affordable (as most traditional healers are related to them in some way in the village*, *for example*, *someone’s uncle and easily reached) than going to the hospital*, *which is costly*, *for example*, *may require transportation to reach a hospital…*.*"* Clinician at one of the health centers/dispensaries

"*…some believe they have been bitten by the snake sent through witchcraft*, *therefore*, *believe traditional healers are more efficient in treating them than hospitals…*.*"* Nurse at one of the health centers/dispensaries

In survey data, seventeen percent (n = 18) of the HCWs believe that traditional herbs help to manage snakebites (S4 Table).

### Knowledge about the administration of SAV

Among the nurses, twelve (26%) said that they had ever administered SAV Among the clinicians, eighteen (42%) said they had ever prescribed SAV (S5 Table). Of the 30 clinicians and nurses, 50% said they prescribe or administer SAV based on the patient’s presentation and type of snake,15% said they give one vial, 12% reported giving between 2 and 3 vials, whereas 18% said others and only 1 HCW reported not remember. Providing the correct prescription was reported to be challenging:

"*…I just wrote give snake antivenom; I believe nurses gave the right dose in consultation with the pharmacist…*.*"* Clinician at one of the hospitals

"*…usually*, *I don’t write the actual dose*, *I just write give snake antivenom*, *the dosing is done in the wards…*.*"* Clinician at one of the hospitals

Most HCWs (n = 84, 80%) did not know the composition of SAV, and only 21 HCWs (20%) reported that it is made of proteins. Seventy-two percent (n = 76) knew that SAV is the standard treatment for envenoming. Eighty-five percent (n = 89) of the HCWs said that SAV can cause a severe hypersensitivity reaction. Regarding administration, 71% of participants reported that it cannot be given orally, with 82%, 62%, and 22% reporting SAV could be given intravenously, intramuscularly, or intradermally, respectively. Fifty-nine percent of the HCWs (n = 62) reported that SAV needs to be reconstituted before use and 24% (n = 25) said that a tourniquet should be applied before the administration as part of the first aid. A polyvalent snake antivenom (Snake Venom Antiserum African-IHS) by VINS Bioproducts Ltd was only available at the Lisungwi Community hospital and Neno District Hospital and in stock at the time of the survey (S5 Table).

### Health care workers’ knowledge of snake identification

Significant gaps in knowledge exist regarding snake identification. Sixty-four percent of the HCWs could name and identify Puff adder [*Bitis arietans*], 61% the Black mamba [*Dendroaspis polylepis*], and 56% the Mozambique spitting cobra [*Naja mossambica*]. Sixty-five percent, 63% and 67% respectively, correctly identified these snakes as venomous species. For the Common house snake [*Boaedon capensis*], Oates’ vine twig [*Thelotornis oastesi*], and spotted bush snake [*Philothamnus semivariegatus*], the majority of > 90% did not know the name and did not correctly identify the snake as venomous or non-venomous (Table 2).

**Table 2:** Health care workers’ knowledge of snake names and their venom status.

N = 84	Identified snake name			Venomous status		
n (%)	Correct	Not correct	Don’t know	Venomous	Non-venomous	Don’t know
Puff adder^1^	54 (64.0)	5 (6.0)	25 (30.0)	55 (65.0)	2 (2.4)	27 (32.0)
Black mamba^2^	51 (61.0)	3 (3.6)	30 (36.0)	53 (63.0)	1 (1.2)	30 (36.0)
Common house snake^3^	2 (2.4)	5 (6.0)	77 (92.0)	6 (7.1)	2 (2.4)	76 (90.0)
Oates’ vine twig^4^	2 (2.4)	2 (2.4)	80 (95.0)	3 (3.6)	2 (2.4)	79 (94.0)
Spotted bush snake^5^	2 (2.4)	16 (19.0)	66 (79.0)	13 (15.0)	3 (3.6)	68 (81.0)
Mozambique spitting cobra^6^	47 (56.0)	8 (9.5)	29 (35.0)	56 (67.0)	1 (1.2)	27 (32.0)

^1^Bitis arietans; local names: Mphiri, Mpiri, Chiphiri, and Cipiri.

^2^Dendroaspis polylepis, commonly called mamba.

^3^Boaedon capensis; local names: Chankusa, Chakusa.

^4^Thelotornis oastesi; local names: Nakalikukuti, Kalikukuti or Kamutimuti.

^5^Philothamnus semivariegatus; local names: Camasamba, Cipota Masamba, Njokansipu and Namasamba.

^6^Naja mossambic; local names: Mamba and Citawo.

### Register review of Snakebite cases in Neno District

We reviewed 185 snakebite cases (36 per 100,000 population) documented in outpatient and inpatient registers from 2018 to 2021 in Neno District. For victim demographics, there were slightly more females compared to males (n = 98, 52.9%) with an overall median age of 25.8 years (IQR = 10–68) (Table 3).

**Table 3:** Snakebite cases reported to health facilities between Jan. 2018 to Dec. 2021.

		Sex of snakebite victim	
Variable	Overall, N = 185	Female, N = 98	Male, N = 87
Age (years)^1^	22.0 (14.0, 36.0)	21.5 (13.2, 36.8)	23.0 (14.0, 35.0)
Age categorized (years)			
<18	71 (38%)	36 (37%)	35 (40%)
18–49	94 (51%)	51 (52%)	43 (49%)
50+	20 (11%)	11 (11%)	9 (10%)
Health facility presented first			
Health Centre/dispensary	59 (32%)	31 (32%)	28 (32%)
Hospital	126 (68%)	67 (68%)	59 (68%)
Season snakebite occurred			
Dry season	65 (35%)	35 (36%)	30 (34%)
Rainy season	120 (65%)	63 (64%)	57 (66%)
Year received care			
2018	38 (21%)	18 (18%)	20 (23%)
2019	49 (26%)	26 (27%)	23 (26%)
2020	54 (29%)	27 (28%)	27 (31%)
2021	44 (24%)	27 (28%)	17 (20%)
Type of care^3^			
in-patient	97 (52%)	53 (54%)	44 (51%)
out patient	88 (48%)	45 (46%)	43 (49%)
Number of days as in-patient (n = 97)			
<3	70 (72%)	38 (72%)	32 (73%)
3–5	22 (23%)	13 (25%)	9 (20%)
>5	5 (5.2%)	2 (3.8%)	3 (6.8%)
Outcome			
Alive	176 (95%)	95 (97%)	81 (93%)
Died	2 (1.1%)	0 (0%)	2 (2.3%)
Referred to QECH^2^	7 (3.8%)	3 (3.1%)	4 (4.6%)

^1^Median (IQR)

^2^QECH = Queen Elizabeth Central Hospital

^3^SAV is available only at 2 hospitals, hence victims that require it may be likely treated as an in-patient

**Fig 2:**
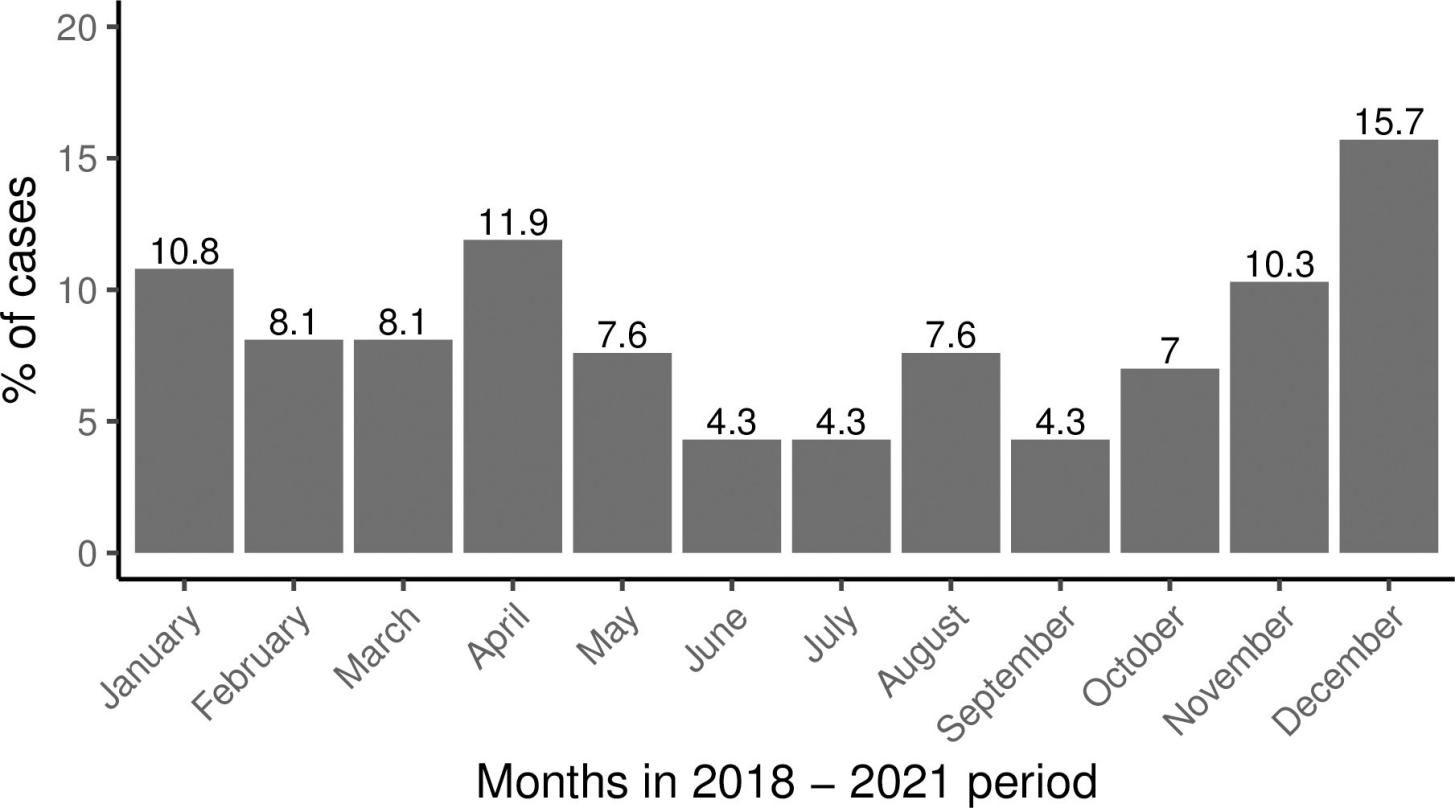
The proportion of snakebite cases managed and treated in Neno District Malawi.

Fifty-one percent of the cases (n = 94) were aged 18–49 years, and children accounted for 38% of the snakebites (n = 71). Sixty-eight percent of the cases (n = 126) were treated at the two hospitals where SAV was available, while the rest were managed at various health centers across the district. There were more cases recorded in the rainy season compared to the dry season (n = 120, 65%), with the highest number in December (15.7%) (Fig 2).

**Fig 3:**
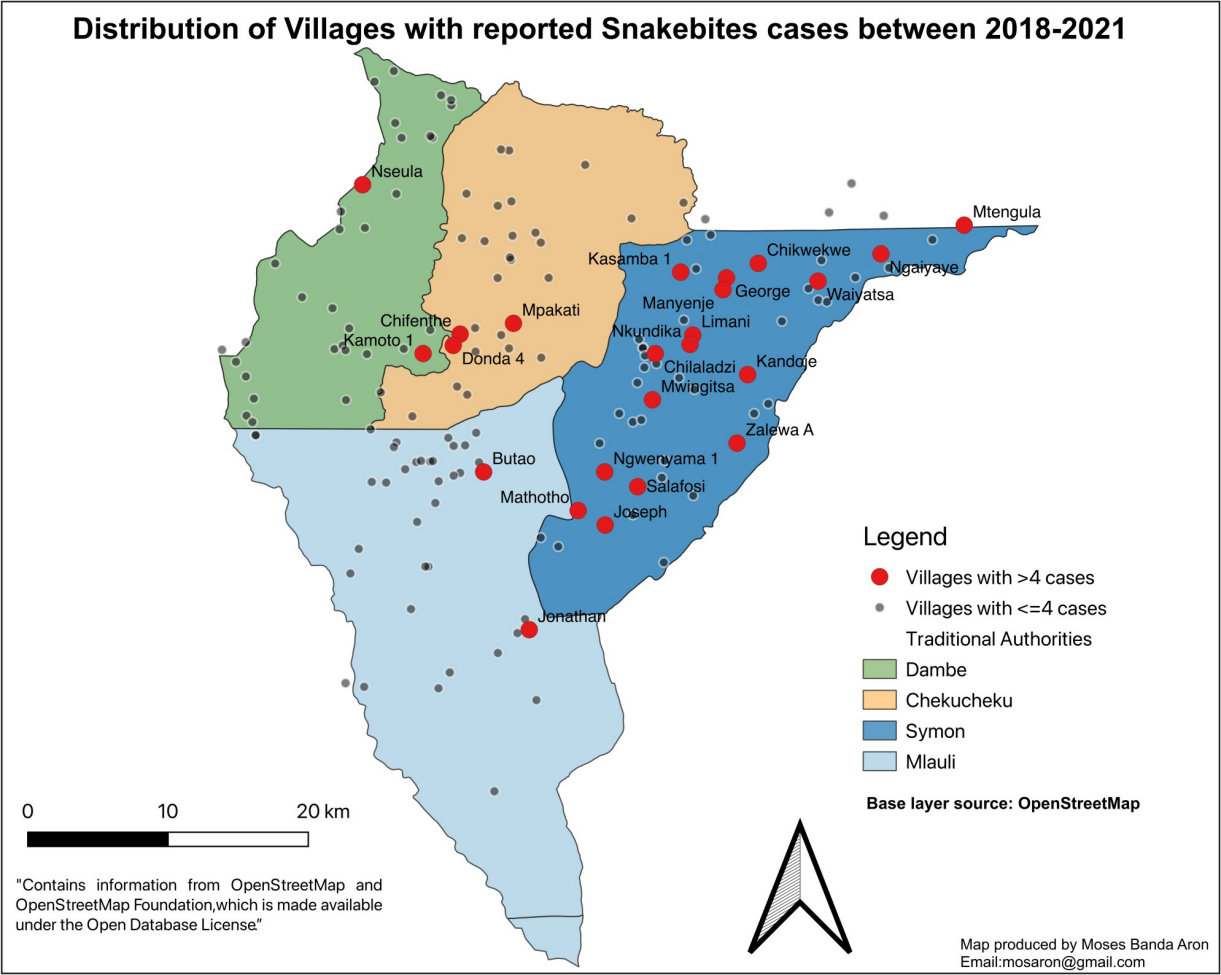
Map of villages where snakebite cases occurred in the Neno District between 2018–2021. We obtained Base map and data from OpenStreetMap and OpenStreetMap Foundation repository [45] and extracted Neno District boundaries.

The least number of snakebite cases (4.3%) was recorded in June, July, and September. Out of 97 patients who were treated as an inpatient, 72% spent less than three days in the hospital, and only 5.2% (n = 5) spent more than five days. Overall, two patients died, seven were referred to Queen Elizabeth Central Hospital (QECH) in Blantyre, Malawi (outcome not known), and the rest were discharged alive. The snakebite cases occurred in almost all villages in the district. However, Traditional Authority (T/A) Symon in the dry flat eastern part of the district had several villages with more than 4 cases of snakebites (Fig 3).

## Discussion

This is the first study in Malawi assessing HCWs’ knowledge of snakebite management. We found significant gaps in knowledge regarding snake identification and snakebite management among HCWs despite receiving pre-service training, suggesting the need for additional knowledge-building opportunities. HCWs reported that snakebite victims go to traditional healers instead of or before going to the health facility for reasons ranging from cost to availability of SAV and cultural beliefs. We also found that more snakebites occurred in the rainy season, that over one-third of the victims are children aged less than 18 and that over half of the cases presented were treated as inpatients.

Our study found that most HCWs considered snakebite a problem in Neno District. However, guidelines for treating or managing snakebite in health facilities were unavailable. Similar findings have been reported in studies done in Ghana and India, where, despite the availability of national guidelines, not all facilities had them ready [9,46]. Availability, quality, and efficacy of SAVs are key to reducing mortality related to snakebite. At the time of the survey, a polyvalent snake antivenom (African IHS) produced by VINS Bioproducts Ltd was available at the two hospitals in the Neno District through the support of Partners In Health, an international non-governmental organization. However, the product’s efficacy against venomous snakes in the region has not been assessed in-vivo. Other hospitals in Malawi, including tertiary level facilities, reported a total lack of these lifesaving drugs [35].

A recent study [47] analyzed 16 antivenom products in African markets, with sobering results. Three products were tested in robust clinical studies and found to be effective against the venom of the West African carpet viper (*Echis ocellatus*), which is not found in Malawi. Four products were evaluated only in observational single-arm studies with varying results. For nine other products, there are either no data in the public domain or data suggesting a lack of effectiveness. There are also no data on efficacy available for Snake Venom Antiserum African-IHS produced by VINS Bioproducts, which is available in the Neno District. The WHO is in the process of reviewing available antivenom products and giving urgent recommendations for necessary action. To develop locally adapted clinical guidelines, a more in-depth understanding of the epidemiology of snakebites in the district and data on the efficacy of available antivenom is urgently needed.

We found significant knowledge gaps regarding snake identification among HCWs. For the set of venomous snakes, except Oates’ Vine snake (twig snake), two-thirds of HCWs could name and identify the snake correctly. The majority of respondents (> 90%) didn’t know the name and species of non-venomous snakes. This could be attributed to the Malawian training curriculum, which offers general training of the HCW on snakebite management but, in our experience, does not include identification of snakes. In addition, there is barely any printed material on snake identification available to the public in Malawi. An evaluation study done in Nepal [48] found that there was a lack of educational materials to aid HCWs to identify snakes, indicating that the problem is not unique to Malawi. The availability of materials could improve the knowledge of HCWs in this respect. However, it is not common in Malawi for snakebite victims to bring the snake that has bitten them to the hospital and photos were rarely available in the past. This is slowly changing with the increased distribution of smartphone cameras indicating the importance of creating training materials and making them available to HCWs. The lack of knowledge has severe implications for the quality of treatment and outcomes, and the likelihood of using SAV where it is not indicated remains high. Lack of knowledge among HCWs on snake identification has also been reported in South Africa, Bangladesh, Lao PDR, and Hong Kong [29,49–51]. A scoping review of studies from 35 countries on snakebite practices of communities and health care providers found that in over 100 cases, snakes were misidentified as venomous species but were actually non- or mildly venomous [52]. There are also reports from Nepal [53], where antivenom was used to treat proven non-venomous snakebite cases. The knowledge of HCWs on snakes and their identification needs to be improved in Neno District to enable proper diagnosis and treatment.

Furthermore, knowledge regarding prescription and antivenom administration was relatively poor among HCWs, with less understanding among "clinicians" than nurses. Similar findings have been reported in Ghana, where HCWs overestimated their knowledge about snakebite management [24], and in Nigeria, where the knowledge about SAV among HCWs was grossly inadequate [28]. Patterns of lack of knowledge among HCWs on snakebite and SAV prescription and administration have also been reported in Brazil, Palestine, Kenya, Uganda, and Zambia [16,18,33]. While over two-thirds of the HCWs said that they had received training on snakebite management, this mainly was during their pre-service training at school, and it was unclear how the training was conducted. High-quality pre-service training with continued on-job training of the HCWs should be considered, as it has proven to improve the health outcomes of snakebite victims in many settings [15,24]. In addition, the Ministry of Health should consider a consultancy service on snakebite identification and advice on management. Close to one-third of HCWs said that a tourniquet should be applied as first aid for snakebite, however, there is extensive literature discouraging use of the tourniquet as a pre-hospital intervention [17,54]. There was no observable difference in knowledge between HCWs with different levels of experience. This indicates the need to improve training in the management of snakebites both the initial training, as well as during continuous professional development. To improve training of HCWs, locally adapted curricula should be developed, based on guidelines reflecting the local circumstances, again indicating the need for higher quality data on the local epidemiology of snakebites.

Compared to other Sub-Saharan African countries and settings like Nigeria, Cameroon, and Ghana, we found health institution-based prevalence of snakebite to be lower. However, this could be an underestimation due to poor documentation in Malawian health facilities [55]. Similarly, we found most HCW believe that snakebite victims prefer being treated by traditional healers with concerns for the affordability of care and unavailability of SAVs along with cultural beliefs on treatment. Similar findings have been widely reported in South America, Asia, and other sub-Saharan African countries [19,21,23,35,56,57]. It has been shown that raising community awareness and availability of SAV in the facilities contribute to a change in health-seeking behavior among snakebite victims. In addition, fostering collaboration between the traditional healer and HCWs is recommended by the World Health Organization [58]. More research is needed in Neno to understand the community member’s knowledge, perception, health seeking behavior and belief on snakebite envenoming and their preference for traditional medicine over “western” bio-medicine.

We found that more snakebites occurred in the rainy season. This finding is not surprising, with >95% of Neno residents being subsistence farmers, mainly in the fields from November to April. They engage in daily manual activities such as vegetation clearing, soil tilling, crop planting, and harvesting, which exposes them to snakes. Studies done in Brazil, Sri Lanka, Ghana, Nigeria, and other countries found that the occurrence of snakebites was seasonal [6,41,59–64]. In addition, snakes prefer dry places, typically houses, during the rainy season, bringing them closer to human beings. Malawi’s temperatures are considerably lower between late April to early September, leading to less snake activity. This has been reported in studies done in Brazil and India showing that snakes prefer high temperatures and a dry climate with little rainfall [65–67]. At the same time, this could partly explain why there are more cases on the eastern side of Neno in Traditional Authority Symon, where temperatures are high with less rainfall over the year. Other possible reasons could be that the area has a lot of goats and cattle which means more headers, the shrub type vegetation, and flat terrain, as well as easier access to the health facilities.

Over half of the snakebite victims in Neno were managed as inpatients at Neno District and Lisungwi community hospitals. They were treated as an inpatient, mostly because SAVs were only available at these two secondary-level hospitals. We also found that over one-third of the cases were children aged less than 18 years. Studies done in Nepal and Nigeria showed that snakebite in children could have higher mortality, owing to the lower volume of distribution relative to the amount of venom injected [68,69]. Case fatality in our study was low (<2%), which is in line with many findings from studies done in tropical and subtropical countries [70–72], and none of those who died were aged less than 18 years. We found a slightly high number of female snakebite victims, which may be due to a higher exposure during daily activities such as fetching water, firewood and farming that usually happen early in the morning or late in the evening. Although not much is known about the aftermath of snakebite victims in Neno, studies conducted in other countries have reported that victims suffer permanent or long-term sequelae, ranging from disability and post-traumatic stress disorder to disadvantaged economic well-being [10,57,61,73–76]. Further studies are required to review and establish the clinical and epidemiological profile of snakebite cases in the district.

This first study on snakebites in Malawi demonstrates that there are significant knowledge gaps among HCWs regarding prescription and administration of SAV and identification of snake species, but also demonstrates how little is known about the epidemiology, referral pathways and clinical consequences of snakebites in Malawi. However, our study had several limitations. First, the study was conducted in a small rural part of Malawi, and its results may not be applicable to urban settings or other rural districts. Nonetheless, the findings are similar to those from other countries and will help to close the data gap on snakebite envenoming in Malawi and southern Africa. Second, during the identification of snakes on pictures, the primary dialect and the ethnic group of the HCWs may have influenced the results as not all would know the Chichewa or English name of the snakes. Similarly, the; dark-colored snakes are often misidentified as mamba or cobra, which may have led to a higher identification rate of the Black Mamba or the Mozambique Spitting Cobra. Identification rates may actually lie even lower than estimated in this study. Further, while we assessed healthcare workers’ knowledge of the composition and application of SAV, our survey lacked questions to identify situations in which antivenom would have been administered. Therefore, it remains unknown whether health care workers would apply SAV even in the case of missing signs of envenomation. However, based on the lack of treatment guidelines, the poor knowledge of general snakebite management and snake identification, as well as anecdotal reports, we assume that there is a high risk of inadequate prescription of SAV, further emphasizing the need for training and development of guidelines. Lastly, the retrospective analysis of register data not primarily designed for research, is another limitation in terms of the level of analysis that could be done. A prospective collection of data on patients presenting with snakebites could address these limitations and should be conducted to address existing knowledge gaps.

## Supporting information

S1 STROBE Checklist The STROBE checklist. (DOCX)

S1 Table Health Care workers Questionnaire. (DOCX)

S2 Table Pictures of Common Venomous and Non-Venomous snakes in Southern Malawi. (DOCX)

S3 Table Snakebite cases tracker for inpatient and outpatient, Neno, Malawi. (DOCX)

S4 Table Health care workers’ knowledge about snakebite treatment, complications and fatality. (DOCX)

S5 Table Knowledge of SAV administration among health care workers. (DOCX)
