# Supplementary material for: Health care workers’ knowledge on identification, management and treatment of snakebite cases in rural Malawi: A descriptive study
Source: PLoS Negl Trop Dis. 2022 Nov 21;16(11):e0010841. doi: 10.1371/journal.pntd.0010841 (PMC9678285; doi:10.1371/journal.pntd.0010841)
Supplement: S1 STROBE Checklist — (DOCX) [file pntd.0010841.s001.docx]

**S1 The STROBE Checklist**

|  | Item No | Recommendation |
| --- | --- | --- |
| **Title and abstract** | 1 | (*a*) Indicate the study’s design with a commonly used term in the title or the abstract **- the study design is indicated at the title** |
|  |  | (*b*) Provide in the abstract an informative and balanced summary of what was done and what was found - **an abstract is presented at the first page of the manuscript** |
| Introduction | | |
| Background/rationale | 2 | Explain the scientific background and rationale for the investigation being reported - **an introduction section is presented after the abstract** |
| Objectives | 3 | State specific objectives, including any prespecified hypotheses **– the objectives of the study are presented at the end of the introduction section** |
| Methods | | |
| Study design | 4 | Present key elements of study design early in the paper – **the study design is presented as the first paragraph of the Methods section** |
| Setting | 5 | Describe the setting, locations, and relevant dates, including periods of recruitment, exposure, follow-up, and data collection **- the setting, study location, the dates the study was done, cases of snakebite reviewed, sampling and data collection are presented at the Methods section** |
| Participants | 6 | (*a*) Give the eligibility criteria, and the sources and methods of selection of participants **– the eligibility is stated at the study population within the Methods section** |
| Variables | 7 | Clearly define all outcomes, exposures, predictors, potential confounders, and effect modifiers. Give diagnostic criteria, if applicable – **a “Data collection and data analysis” is presented at the Methods section** |
| Data sources/ measurement | 8* | For each variable of interest, give sources of data and details of methods of assessment (measurement). Describe comparability of assessment methods if there is more than one group - **a “Data collection and data analysis” is presented at the Methods section** |
| Bias | 9 | Describe any efforts to address potential sources of bias - **a “Data collection and data analysis” is presented at the Methods section** |
| Study size | 10 | Explain how the study size was arrived at **- a ‘study population’ section is presented at Methods** |
| Quantitative variables | 11 | Explain how quantitative variables were handled in the analyses. If applicable, describe which groupings were chosen and why - **a ‘Data analysis’ section is presented at Methods** |
| Statistical methods | 12 | (*a*) Describe all statistical methods, including those used to control for confounding **- a ‘Data analysis’ section is presented at Methods** |
|  |  | (*b*) Describe any methods used to examine subgroups and interactions **- a ‘Data analysis’ section is presented at Methods** |
|  |  | (*c*) Explain how missing data were addressed **- a ‘Data analysis’ section is presented at Methods** |
|  |  | (*d*) If applicable, describe analytical methods taking account of sampling strategy **- a ‘Data analysis’ section is presented at Methods** |
|  |  | (*e*) Describe any sensitivity analyses **- a ‘Data analysis’ section is presented at Methods** |
| Results | | |
| Participants | 13* | (a) Report numbers of individuals at each stage of study—eg numbers potentially eligible, examined for eligibility, confirmed eligible, included in the study, completing follow-up, and analysed – “**Socio-demographic characteristics” section is presented at Results** |
|  |  | (b) Give reasons for non-participation at each stage - “**Socio-demographic characteristics” section is presented at Results** |
|  |  | (c) Consider use of a flow diagram **Not used because already presented in the methods section and also Socio-demographic characteristics section presented at Results** |
| Descriptive data | 14* | (a) Give characteristics of study participants (eg demographic, clinical, social) and information on exposures and potential confounders - “**Socio-demographic characteristics” section is presented at Results** |
|  |  | (b) Indicate number of participants with missing data for each variable of interest - “**Socio-demographic characteristics” section is presented at Results** |
| Outcome data | 15* | Report numbers of outcome events or summary measures – **Sections on snakebite training and experiences among health care workers, knowledge about administration of SAV, knowledge of snake identification and register review of snakebite cases are included in the results** |
| Main results | 16 | (*a*) Give unadjusted estimates and, if applicable, confounder-adjusted estimates and their precision (eg, 95% confidence interval). Make clear which confounders were adjusted for and why they were included – **Not used as the level of the analysis was purely descriptive** |
|  |  | (*b*) Report category boundaries when continuous variables were categorized - **Not applicable** |
|  |  | (*c*) If relevant, consider translating estimates of relative risk into absolute risk for a meaningful time period **- Not applicable** |
| Other analyses | 17 | Report other analyses done—eg analyses of subgroups and interactions, and sensitivity analyses - **Not applicable** |
| Discussion | | |
| Key results | 18 | Summarise key results with reference to study objectives - **section of ‘study results summary’ is present as first paragraph of the Discussion** |
| Limitations | 19 | Discuss limitations of the study, taking into account sources of potential bias or imprecision. Discuss both direction and magnitude of any potential bias **Section of ‘limitations’ is present as last paragraph of the Discussion** |
| Interpretation | 20 | Give a cautious overall interpretation of results considering objectives, limitations, multiplicity of analyses, results from similar studies, and other relevant evidence **See Discussion section at all** |
| Generalisability | 21 | Discuss the generalisability (external validity) of the study results - **see Discussion section at all** |
| Other information | | |
| Funding | 22 | Give the source of funding and the role of the funders for the present study and, if applicable, for the original study on which the present article is based **- A ‘Funding’ section is presented after ‘Ethical Consideration’** |

*Give information separately for exposed and unexposed groups.

**Note:** An Explanation and Elaboration article discusses each checklist item and gives methodological background and published examples of transparent reporting. The STROBE checklist is best used in conjunction with this article (freely available on the Web sites of PLoS Medicine at http://www.plosmedicine.org/, Annals of Internal Medicine at http://www.annals.org/, and Epidemiology at http://www.epidem.com/). Information on the STROBE Initiative is available at www.strobe-statement.org.
