# Supplementary material for: Health care workers’ knowledge on identification, management and treatment of snakebite cases in rural Malawi: A descriptive study
Source: PLoS Negl Trop Dis. 2022 Nov 21;16(11):e0010841. doi: 10.1371/journal.pntd.0010841 (PMC9678285; doi:10.1371/journal.pntd.0010841)
Supplement: S1 Table — (DOCX) [file pntd.0010841.s002.docx]

**S1 Table. Health Care workers Questionnaire**

| Section A. Socio-demographic information | | | |
| --- | --- | --- | --- |
| Sn | Question | Options | Skip |
| A1. | Sex | 1. Male 2. Female |  |
| A2. | Age | \|___\|___\| Years  99. Don’t Know |  |
| A3. | Professional qualification | 1. Clinician (MA, CO) 2. Nurse (all cadres) 3. Pharm. Technician /Pharmacist 4. Medical Doctor (MBSS/MD/DO) |  |
| A5 | Where did you spend most of your childhood | 1. Rural 2. Urban |  |
| A6 | Place of practice | 1. Health Centre / Dispensary 2. Hospital |  |
| A7 | Number of years of practice (to the nearest whole number) | ___________________________ |  |
| Section B. Knowledge of SAV, dosage, and mode of administration | | | |
| B1. | Have you ever administered or prescribed SAV to snakebite a victim? | 1. Yes 2. No 3. Don’t know |  |
| B2. | What would be the appropriate dose of SAV for an adult victim? | 1. 1 vial 2. 2-3 vials 3. Based on the patient’s presentation and type of snake 4. Other 5. Don’t know |  |
| B3 | Explain how you do the SAV prescription or the administration  (Depending on HCW) | ______________________________ |  |
| B4. | What is the major component of SAV | 1. Carbohydrate 2. Protein 3. Fat 4. Heavy metal 5. Don’t know |  |
| B6. | SAV is the only standard treatment for envenoming | 1. Yes 2. No 3. Don’t know |  |
| B7. | SAV can cause a severe hypersensitivity reaction | 1. Yes 2. No 3. Don’t know |  |
| B8. | SAV can be administered orally | 1. Yes 2. No 3. Don’t know |  |
| B9. | SAV can be administered intravenously | 1. Yes 2. No 3. Don’t know |  |
| B10. | SAV is preferably administered intramuscularly | 1. Yes 2. No 3. Don’t know |  |
| B11. | SAV is preferably administered intradermally | 1. Yes 2. No 3. Don’t know |  |
| B12. | All forms of SAV need to be reconstituted before use | 1. Yes 2. No 3. Don’t know |  |
| B13. | The tourniquet should be applied before the administration of SAV as first aid | 1. Yes 2. No 3. Don’t know |  |
| B14 | In the last 6 months, has your facility distributed SAV? | 1. Yes 2. No 3. Don’t know |  |
| B15 | Does your facility have stock of SAV | 1. Yes 2. No 3. Don’t know |  |
| B16 | Have you ever been trained in snakebite management? | 1. Yes 2. No 3. Don’t know | If no skip to B8 |
| Section C. Snakebite treatment and management | | | |
| C1 | Do you think snake is problem in Neno district | 1. Yes 2. No 3. Don’t know |  |
| C2 | Give a reason for your answer | _____________________________ |  |
| C3 | Have you ever been trained in snakebite management | 1. Yes 2. No 3. Don’t know |  |
| C4. | If YES above, what was the medium of  'training (you can tick more than one option) | 1. Workshop 2. Textbooks 3. Senior colleague 4. Leaflets 5. Posters 6. At school |  |
| C5 | Does your facility have a protocol for the management of snakebite? | 1. Yes 2. No 3. Don’t know |  |
| C6 | What do you do when people report to your health facility with snakebite? | 1. Refer immediately 2. Give first aid treatments and refer 3. Admit and treat 4. Call for assistance from another health facility |  |
| C7 | In the past year, have you treated/managed any snake bite | 1. Yes 2. No 3. Don’t know | If no skip to C13 |
| C8 | About how many snakebite cases do you get in a year? | 99. Don’t know/can’t remember |  |
| C9 | Which part of the body receives the most bites? | 1. Legs 2. Hands 3. Trunk 4. Head 5. Other 6. Don’t know 7. Specify_____________________ |  |
| C10 | Which time of the day do you receive more snakebite cases? | 1. Morning 2. Afternoon 3. Evening/Night 4. Don’t know |  |
| C11 | What could be the causes of the snakebite complications that do occur at your health Centre/hospital? | 1. Delay by victims to report for treatment 2. Other drastic first-aid measures used by victims 3. Mistakes in the application of treatment 4. Inefficiency of your treatment 5. Others |  |
| C12 | Have you recorded any fatalities in snake bite at your health Centre/hospital? | 1. Yes 2. No 3. Don’t know |  |
| C13 | Do you think snakebite victims visit local healers before they reach the hospital? | 1. Yes 2. No 3. Don’t know |  |
| C14 | If yes, why do you think they do that? | ____________________________ |  |
| C15 | Do you think that traditional herbs help manage snakebites | 1. Yes 2. No 3. Don’t know |  |
| Section D. Snake Identification (Venomous vs non-Venomous)  Instruction: Ask the interviewee if it is okay to show him/ her pictures of snakes for identification | | | |
| Sn | Question | Options | Skip |
| D1 | Are you comfortable seeing pictures of snakes? | 1. Yes 2. No | If no, end the survey |
| D3. | What is the name of the snake labelled A? | 99. Don’t know |  |
| D4. | Is the snake venomous? | 1. Yes 2. No |  |
| D5 | What is the name of the snake labelled B | 99. Don’t know |  |
| D6. | Is the snake venomous | 1. Yes 2. No |  |
| D7. | What is the name of the snake labelled C? | 99. Don’t know |  |
| D8. | Is the snake venomous? | 1. Yes 2. No |  |
| D9 | What is the name of the snake labelled D | 99. Don’t know |  |
| D10. | Is the snake venomous | 1. Yes 2. No |  |
| D11 | What is the name of the snake labelled E? | 99. Don’t know |  |
| D12. | Is the snake venomous? | 1. Yes 2. No |  |
| D13 | What is the name of the snake labelled F | 99. Don’t know |  |
| D14 | Is the snake venomous | 1. Yes 2. No |  |
| Thank you for your participation | | | |
