# Supplementary material for: Health care workers’ knowledge on identification, management and treatment of snakebite cases in rural Malawi: A descriptive study
Source: PLoS Negl Trop Dis. 2022 Nov 21;16(11):e0010841. doi: 10.1371/journal.pntd.0010841 (PMC9678285; doi:10.1371/journal.pntd.0010841)
Supplement: S2 Table — (DOCX) [file pntd.0010841.s003.docx]

**S2 Table. Pictures of Common Venomous and Non-Venomous snakes in Southern Malawi**

| Puff Adder (venomous)  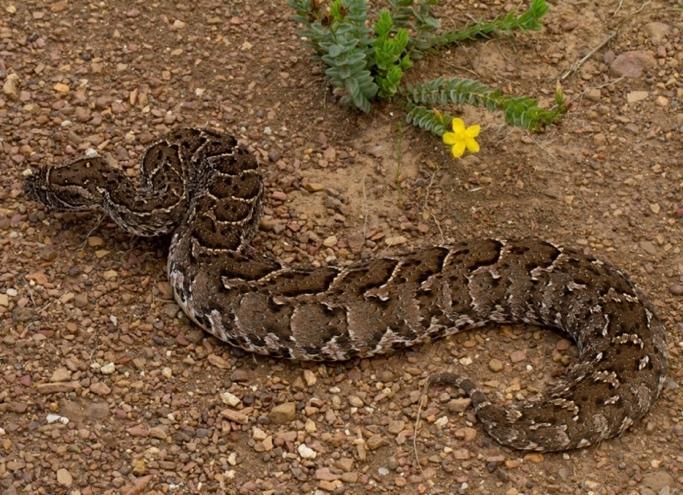 | Black Mamba (venomous)  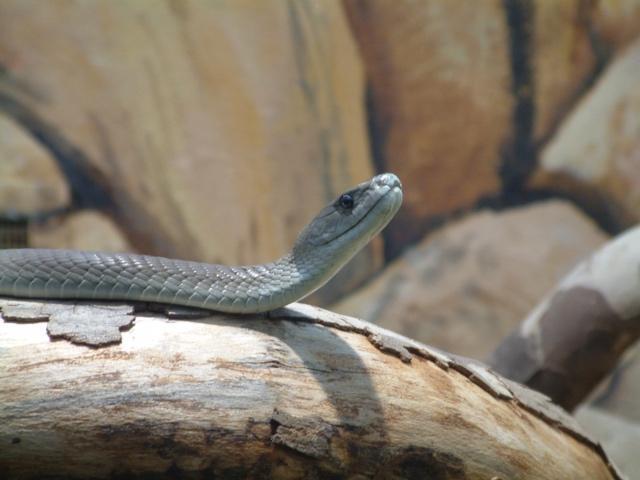 |
| --- | --- |
| Common House Snake (non-venomous)  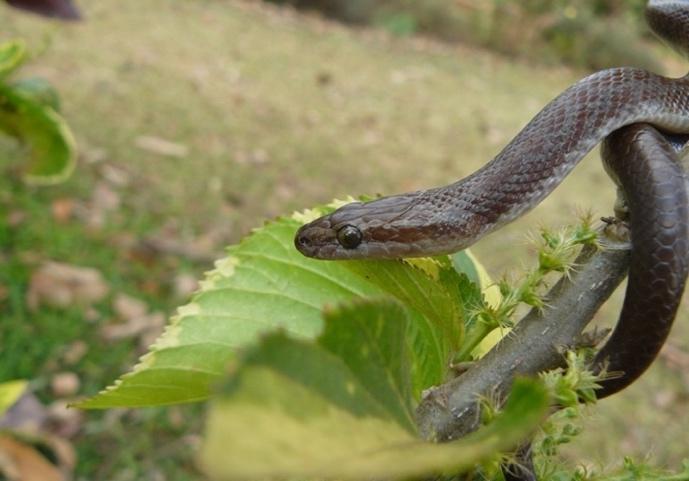 | Oates’ vine twig (venomous)  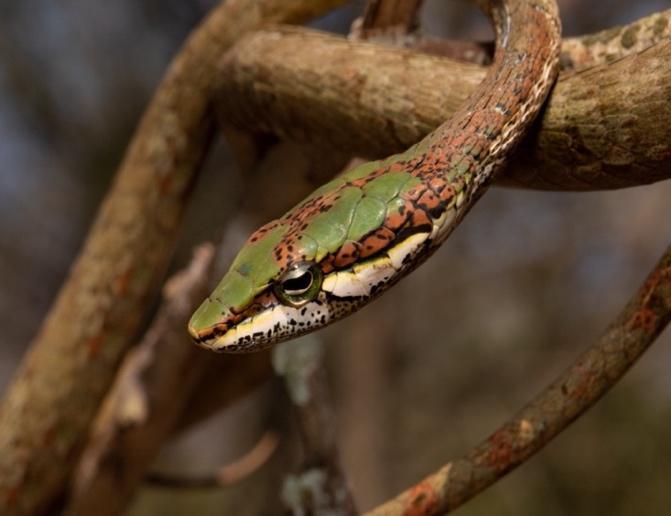 |
| Spotted bush snake (non-venomous)  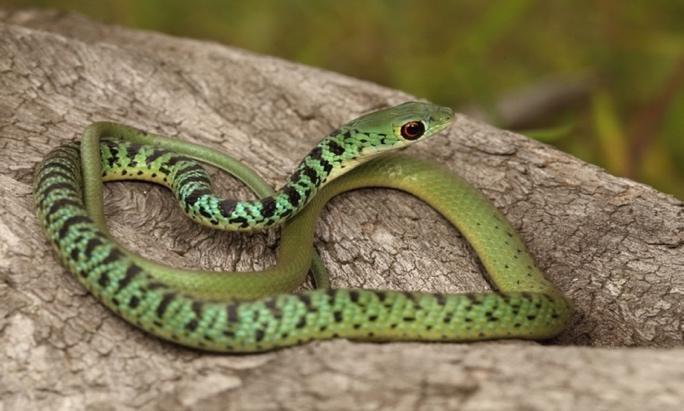 | Mozambique Spitting Cobra (venomous)  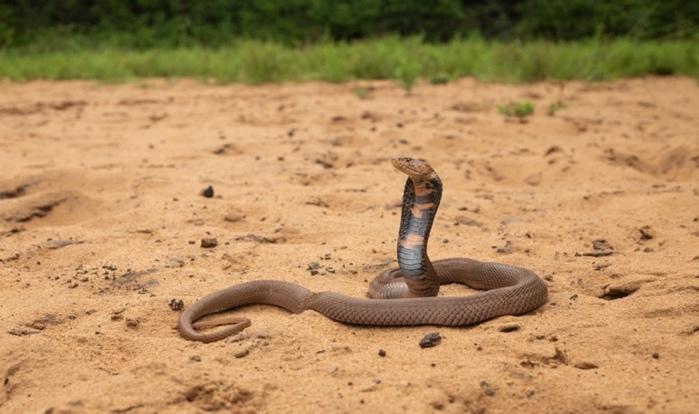 |
