# Supplementary material for: Health care workers’ knowledge on identification, management and treatment of snakebite cases in rural Malawi: A descriptive study
Source: PLoS Negl Trop Dis. 2022 Nov 21;16(11):e0010841. doi: 10.1371/journal.pntd.0010841 (PMC9678285; doi:10.1371/journal.pntd.0010841)
Supplement: S3 Table — (DOCX) [file pntd.0010841.s004.docx]

**S3 Table. Snakebite cases tracker for inpatient and outpatient, Neno, Malawi**

| Sn | Health facility | Month | Year | village | T/A | Age | Sex  (m/f /not indicated) | Care Type (OPD / In) | Outcome (Alive, died, not indicated) | Number of days – Inpatient only |
| --- | --- | --- | --- | --- | --- | --- | --- | --- | --- | --- |
| 1 |  |  |  |  |  |  |  |  |  |  |
| 2 |  |  |  |  |  |  |  |  |  |  |
| … |  |  |  |  |  |  |  |  |  |  |
| n |  |  |  |  |  |  |  |  |  |  |
