# Supplementary material for: Health care workers’ knowledge on identification, management and treatment of snakebite cases in rural Malawi: A descriptive study
Source: PLoS Negl Trop Dis. 2022 Nov 21;16(11):e0010841. doi: 10.1371/journal.pntd.0010841 (PMC9678285; doi:10.1371/journal.pntd.0010841)
Supplement: S4 Table — (DOCX) [file pntd.0010841.s005.docx]

**S4 Table.** **Health care workers' knowledge about snakebite treatment, complications and fatality**

|  | | Profession | | |
| --- | --- | --- | --- | --- |
| Variable | Overall,  N = 105 | Nurses,  N = 47 | Pharmacy, N = 15 | Clinicians, N = 43 |
| Do you think snakebite is problem in Neno | | | | |
| No | 12 (11%) | 7 (15%) | 2 (13%) | 3 (7.0%) |
| Yes | 93 (89%) | 40 (85%) | 13 (87%) | 40 (93%) |
| Have you ever been trained in snakebite management | | | | |
| No | 28 (27%) | 16 (34%) | 9 (60%) | 3 (7.0%) |
| Yes | 77 (73%) | 31 (66%) | 6 (40%) | 40 (93%) |
| Does your facility have a protocol for the management of snakebite? | | | | |
| Don’t know | 25 (24%) | 11 (23%) | 6 (40%) | 8 (19%) |
| No | 78 (74%) | 34 (72%) | 9 (60%) | 35 (81%) |
| Yes | 2 (1.9%) | 2 (4.3%) | 0 (0%) | 0 (0%) |
| What do you do when people report to your health facility with snakebite? | | | | |
| Admit and treat | 65 (62%) | 31 (66%) | 6 (40%) | 28 (65%) |
| Call for assistance from another health facility | 1 (1.0%) | 1 (2.1%) | 0 (0%) | 0 (0%) |
| Give first aid treatments and refer | 35 (33%) | 12 (26%) | 9 (60%) | 14 (33%) |
| Refer immediately | 4 (3.8%) | 3 (6.4%) | 0 (0%) | 1 (2.3%) |
| In the past year, have you treated / managed any snake bite | | | | |
| Don’t know | 1 (1.0%) | 0 (0%) | 1 (6.7%) | 0 (0%) |
| No | 56 (53%) | 31 (66%) | 9 (60%) | 16 (37%) |
| Yes | 48 (46%) | 16 (34%) | 5 (33%) | 27 (63%) |
| About how many snakebite cases do you get in a year? | | | | |
| <5 | 49 (47%) | 21 (45%) | 5 (33%) | 23 (53% |
| 5-15 | 20 (19%) | 7 (15%) | 4 (27%) | 9 (21%) |
| >15 | 1 (1.0%) | 0 (0%) | 1 (6.7%) | 0 (0%) |
| Don’t know | 35 (33%) | 19 (40%) | 5 (33%) | 11 (26%) |
| Which part of the body receives the most bites? | | | | |
| hands | 8 (7.6%) | 5 (11%) | 1 (6.7%) | 2 (4.7%) |
| legs | 97 (92%) | 42 (89%) | 14 (93%) | 41 (95%) |
| Which time do you receive more snakebite cases? | | | |  |
| Afternoon | 12 (11%) | 7 (15%) | 3 (20%) | 2 (4.7%) |
| Don’t know | 9 (8.6%) | 7 (15%) | 1 (6.7%) | 1 (2.3%) |
| Evening / night | 70 (67%) | 29 (62%) | 8 (53%) | 33 (77%) |
| Morning | 14 (13%) | 4 (8.5%) | 3 (20%) | 7 (16%) |
| What could be the most causes of the snakebite complications that do occur at your facility? | | | | |
| Delay by victims to report for treatment | 77 (73%) | 33 (70%) | 11 (73%) | 33 (77%) |
| Inefficiency of treatment | 4 (3.8%) | 1 (2.1%) | 0 (0%) | 3 (7.0%) |
| Mistakes in the application of treatment | 3 (2.9%) | 2 (4.3%) | 0 (0%) | 1 (2.3%) |
| Other drastic first-aid measures used by victims | 17 (16%) | 8 (17%) | 3 (20%) | 6 (14%) |
| others | 4 (3.8%) | 3 (6.4%) | 1 (6.7%) | 0 (0%) |
| Have you recorded any fatalities due to snake bites at your health Centre/hospital last year? | | | | |
| Don’t know | 14 (13%) | 6 (13%) | 3 (20%) | 5 (12%) |
| No | 67 (64%) | 31 (66%) | 9 (60%) | 27 (63%) |
| Yes | 24 (23%) | 10 (21%) | 3 (20%) | 11 (26%) |
| Do snakebite victims visit healers first before visiting the heath facility | | | | |
| Don’t know | 3 (2.9%) | 1 (2.1%) | 0 (0%) | 2 (4.7%) |
| No | 2 (1.9%) | 1 (2.1%) | 0 (0%) | 1 (2.3%) |
| Yes | 100 (95%) | 45 (96%) | 15 (100%) | 40 (93%) |
| Do you think that traditional herbs help manage snakebites | | | | |
| Don’t know | 18 (17%) | 5 (11%) | 2 (13%) | 11 (26%) |
| No | 69 (66%) | 31 (66%) | 9 (60%) | 29 (67%) |
| Yes | 18 (17%) | 11 (23%) | 4 (27%) | 3 (7.0%) |
