# Supplementary material for: Health care workers’ knowledge on identification, management and treatment of snakebite cases in rural Malawi: A descriptive study
Source: PLoS Negl Trop Dis. 2022 Nov 21;16(11):e0010841. doi: 10.1371/journal.pntd.0010841 (PMC9678285; doi:10.1371/journal.pntd.0010841)
Supplement: S5 Table — (DOCX) [file pntd.0010841.s006.docx]

**S5 Table. Knowledge of SAV administration among health care workers**

| Profession | | | | | |
| --- | --- | --- | --- | --- | --- |
| Variable  n (%) | Overall  N = 105 | | Nurses  N = 47 | Pharmacy  N = 15 | Clinicians  N = 43 |
| Have you ever administered or prescribe SAV to snakebite a victim | | | | | |
| No | 60 (69%) | | 35 (74%) | N/A | 25 (58%) |
| Yes | 30 (31%) | | 12 (26%) | N/A | 18 (42%) |
| What would the appropriate dose of SAV for an adult victim^1^ | | | | | |
| Based on patient’s presentation  and type of snake | 15 (50%) | | 7 (58%) | N/A | 8 (44%) |
| Don’t know | 1 (3.3%) | | 0 (0%) | N/A | 1 (5.6%) |
| other | 6 (20%) | | 0 (0%) | N/A | 6 (33%) |
| 1 vial | 4 (13.3%) | | 3 (25%) | N/A | 1 (5.6%) |
| 2-3 vials | 4 (13.3%) | | 2 (17%) | N/A | 2 (11%) |
| What is the major component of SAV | | | | | |
| Don’t know | 84 (80%) | | 39 (83%) | 10 (67%) | 35 (81%) |
| Protein | 21 (20%) | | 8 (17%) | 5 (33%) | 8 (19%) |
| SAV is the only standard treatment for envenoming | | | | | |
| Don’t know | 19 (18%) | | 13 (28%) | 1 (6.7%) | 5 (12%) |
| No | 10 (9.5%) | | 3 (6.4%) | 3 (20%) | 4 (9.3%) |
| Yes | 76 (72%) | | 31 (66%) | 11 (73%) | 34 (79%) |
| SAV can cause a severe hypersensitivity reaction | | | | | |
| Don’t know | 10 (9.5%) | | 9 (19%) | 1 (6.7%) | 0 (0%) |
| No | 6 (5.7%) | | 2 (4.3%) | 1 (6.7%) | 3 (7.0%) |
| Yes | 89 (85%) | | 36 (77%) | 13 (87%) | 40 (93%) |
| SAV can be administered orally | | | | | |
| Don’t know | | 25 (24%) | 10 (21%) | 5 (33%) | 10 (23%) |
| No | | 75 (71%) | 36 (77%) | 8 (53%) | 31 (72%) |
| Yes | 5 (4.8%) | | 1 (2.1%) | 2 (13%) | 2 (4.7%) |
| SAV can be administered intravenously | | | | | |
| Don’t know | 14 (13%) | | 9 (19%) | 1 (6.7%) | 4 (9.3%) |
| No | 5 (4.8%) | | 3 (6.4%) | 0 (0%) | 2 (4.7%) |
| Yes | 86 (82%) | | 35 (74%) | 14 (93%) | 37 (86%) |
| SAV is preferably administered intramuscularly | | | | | |
| Don’t know | 25 (24%) | | 12 (26%) | 3 (20%) | 10 (23%) |
| No | 15 (14%) | | 8 (17%) | 0 (0%) | 7 (16%) |
| Yes | 65 (62%) | | 27 (57%) | 12 (80%) | 26 (60%) |
| SAV is preferably administered intradermally | | | | | |
| Don’t know | 49 (47%) | | 26 (55%) | 5 (33%) | 18 (42%) |
| No | 33 (31%) | | 12 (26%) | 4 (27%) | 17 (40%) |
| Yes | 23 (22%) | | 9 (19%) | 6 (40%) | 8 (19%) |
| All forms of SAV need to be reconstituted before use | | | | | |
| Don’t know | 26 (25%) | | 9 (19%) | 3 (20%) | 14 (33%) |
| No | 17 (16%) | | 8 (17%) | 4 (27%) | 5 (12%) |
| Yes | 62 (59%) | | 30 (64%) | 8 (53%) | 24 (56%) |
| Tourniquet be applied before the administration of SAV as part of first aid | | | | | |
| Don’t know | 29 (28%) | | 16 (34%) | 6 (40%) | 7 (16%) |
| No | 51 (49%) | | 20 (43%) | 4 (27%) | 27 (63%) |
| Yes | 25 (24%) | | 11 (23%) | 5 (33%) | 9 (21%) |
| In the last 6 months, has your facility distributed SAV? | | | | | |
| Don’t know | | | N/A | 1 (6.7%) | N/A |
| No | | | N/A | 9 (60%) | N/A |
| Yes | | | N/A | 5 (33.3%) | N/A |
| Does your facility have stock of SAV | | | | | |
| Don’t know | | | N/A | 1 (6.7%) | N/A |
| No | | | N/A | 9 (60%) | N/A |
| Yes | | | N/A | 5 (33.3%) | N/A |
| ^1^In total 33 HCWs (12 nurses, 2 pharmacy and 18 clinical officers) | | | | | |
